# Polymer Flooding in Heterogeneous Heavy Oil Reservoirs: Experimental and Simulation Studies

**DOI:** 10.3390/polym13162636

**Published:** 2021-08-07

**Authors:** Xiankang Xin, Gaoming Yu, Keliu Wu, Xiaohu Dong, Zhangxin Chen

**Affiliations:** 1 School of Petroleum Engineering, Yangtze University, Wuhan 430100, China; xiankang.xin@hotmail.com; 2 College of Petroleum Engineering, China University of Petroleum, Beijing 102249, China; wukeliu19850109@163.com (K.W.); dongxh@cup.edu.cn (X.D.); 3 Department of Chemical and Petroleum Engineering, University of Calgary, Calgary, AB T2N 1N4, Canada; zhachen@ucalgary.ca

**Keywords:** enhanced oil recovery, heavy oil, polymer flooding, non-Newtonian flow, physical experiment, numerical simulation

## Abstract

Polymer flooding (PF) in heterogeneous heavy oil reservoirs is not only closely related to polymer degradation, but also to non-Newtonian flow. In this paper, both experimental and simulation methods are combined to investigate this type of flooding. Through experiments, the degradation of polymer, rheological properties of fluids, and flow of fluids in porous media were determined. Based on the experimental results, a novel mathematical model was established, and a new PF simulator was designed, validated, and further applied to study the effects of polymer degradation, polymer solution shear thinning, and non-Newtonian flow on PF in heterogeneous heavy oil reservoirs. These experimental results demonstrated that the polymer first-order static degradation rate constant was lower than the polymer first-order dynamic degradation rate constant; the polymer solution and heavy oil were non-Newtonian fluids, with shear thinning and Bingham fluid properties, respectively; and the heavy oil threshold pressure gradient (TPG) in low-permeability porous media was higher than that in high-permeability porous media. All comparison results showed that the designed simulator was highly accurate and reliable, and could well describe both polymer degradation and non-Newtonian flow, with special emphasis on the distinction between polymer static and dynamic degradation and heavy oil TPG. Furthermore, the simulation results verified that polymer degradation, polymer solution shear thinning, and heavy oil TPG all had negative effects on the efficiency of PF in heterogeneous heavy oil reservoirs.

## 1. Introduction

As a source of global economic development, energy has become increasingly prominent as the process of economic globalization continues to accelerate [1]. Crude oil is not only the most important energy source, but also the most critical raw material in modern industrial society [2]. Therefore, the global crude oil demand has become and will become stronger. However, with the continuous exploitation of crude oil resources in recent decades, the production of conventional crude oil resources has decreased, and it is difficult to meet the global crude oil demand [3]. Thus, heavy oil has attracted the attention of many crude oil developers due to its huge geological reserves. Unlike conventional crude oil, heavy oil has high density and high viscosity, which brings great challenges to its exploitation and recovery. Among many enhanced oil recovery (EOR) methods, polymer flooding (PF) has become a commonly used method due to its wide range of field applications. Moreover, it has advantages in supplementing energy and promoting sweep efficiency in heterogeneous heavy oil reservoirs [4]. Therefore, the study of PF in heterogeneous heavy oil reservoirs is necessary to increase the efficiency of heavy oil development and meet the global crude oil demand.

The key to PF momentum is to add water-soluble polymer to water, which increases the viscosity and greatly reduces the fluidity of injected water. Research objectives must work to further maximize the sweep efficiency for producing more heavy oil [5]. PF involves a variety of complex mechanisms, including viscosity alteration, adsorption, degradation, and non-Newtonian flow. These factors can help determine the performance of PF. Therefore, PF is not a simple process of adding polymer to water [6,7]. To obtain a better PF performance, many experimental investigations have been comprehensively studied [8,9], and most of these studies have focused on polymer static degradation [10,11]. However, the study of polymer dynamic degradation in porous media is relatively rare and requires more research. Moreover, from the point of view of heavy oil reservoirs, the non-Newtonian flow of heavy oil can have a great effect on PF efficiency. Several research studies have shown that only when a pressure gradient is higher than the critical threshold pressure gradient (TPG), can heavy oil flow in porous media [12,13,14]. However, a more accurate analysis of the TPG effect on heavy oil is still needed. Various methods were selected for its measurements, such as the steady-state pressure-velocity method, the unstable method, etc. [15,16,17]. However, they involve some drawbacks such as being time-consuming, generating large errors, and difficult data acquisition [18]. Obviously, the heavy oil TPG measurement methods require more attention. Therefore, only through in-depth analysis and research, can better performance and greater advantages of PF be achieved.

Extensive research has proven that PF numerical simulation is a powerful tool for feasibility evaluation, scheme optimization, and production forecasting of PF, which can effectively reduce the risks associated with PF and play a guiding role in assessing and controlling PF [19]. An ideal PF numerical simulator should comprehensively consider the most relevant mechanisms and describe a PF process as accurately as possible in order to assure reliable results [20]. With the continuous development of PF numerical simulators, most of the basic mechanisms have been resolved [7,21,22,23,24,25,26], but polymer degradation and non-Newtonian flow are the exceptions since few PF simulators can handle both polymer degradation and non-Newtonian flow simultaneously. Some methods have been proposed to improve the performance of PF numerical simulators [27,28], but the need is not fulfilled. A concentration decay model and a viscosity decay model are two main mathematical models used for characterizing polymer degradation [29,30]. The former considers that polymer concentration decreases during polymer degradation, while the latter states that only polymer solution viscosity is reduced, but a reduction in polymer concentration has not been addressed as part of the polymer degradation process. In fact, polymer degradation is a process involving the ruptures of long polymer molecular chains, thereby causing a decrease in its average molecular mass rather than its concentration [31,32,33]. Therefore, the concentration decay model is more representative of the nature of polymer degradation. On the contrary, the viscosity decay model describes the variations in polymer solution viscosity over time under static conditions, which can hardly be directly applied to numerical simulators. This is due to the difficulty of obtaining accurate polymer degradation time in simulation grids under dynamic conditions. For non-Newtonian flow, some models have been established to transform a polymer solution flow rate into an equal shear rate, in order to solve the non-Newtonian flow of polymer solutions in porous media [34]. Besides, a modified Darcy’s equation has been introduced to address heavy oil TPG [13]. These methods can provide references for more comprehensive and detailed studies of PF in heterogeneous heavy oil reservoirs via numerical simulations. Unlike experimental research, where a physicochemical property or mechanism can be studied separately, numerical simulation needs to consider all properties comprehensively. Neglecting or failing to consider any variation in flow, shear, and viscoelastic properties, degradation state, or media compressibility can inevitably lead to an inability to accurately describe the entire PF process, causing deviations in simulation results [35]. Based on the premise of polymer degradation and non-Newtonian flow during PF in heterogeneous heavy oil reservoirs, there is an urgent need to establish a simulator that can simultaneously consider them.

In this paper, polymer degradation, rheological properties of polymer solution and heavy oil, and their flow in porous media were investigated by physical experiments. After that, a new mathematical model that can simultaneously consider both polymer degradation and non-Newtonian flow was established. Then, a novel in-house three-dimensional two-phase PF simulator was developed, and it was validated by the industry-standard commercial simulator ECLIPSE (Schlumberger, Houston, TX, USA) [36] and compared with PF experiments. In addition, through the designed simulator in this paper, an in-depth analysis covering the influence of polymer degradation and non-Newtonian flow was fulfilled. The results of this study can provide scientific and effective reference and guidance for enhancing the performance of PF in heterogeneous heavy oil reservoirs.

## 2. Methodology

### 2.1. Physical Experiments

#### 2.1.1. Assessment of Material Properties and Composition

Table 1, Table 2, Table 3 and Table 4 show the information on polymers, brine, heavy oil, and cores. Here, a CG-CF11 rod for thin-layer chromatography (Chuange Sence, Changsha, China) was used to analyze the saturate, aromatic, resin, and asphaltene (SARA) fractions of heavy oil, and a Physica MCR-301 advanced rotary rheometer (Anton Paar, New South Wales, Australia) was applied to measure the viscosity of heavy oil.

#### 2.1.2. Preparation of Polymer Solution

Polymer solution with a concentration of 5 kg/m^3^ was prepared by adding the polymer evenly into the brine. Under the condition of 25 °C the brine was stirred at 120 rpm by a JJ-1B stirrer (Xinrui Instrument Factory, Changzhou, China). The polymer solution was first stirred for 2 h, then deoxidized and sealed in a brown glass bottle for 12 h. After maturation, the polymer mother liquor was obtained and serially diluted with the brine at different concentrations of 2.5, 2, 1.75, 1.5, 1.0, and 0.5 kg/m^3^. The diluted polymer solutions were then stirred at a speed of 100 rpm for 1 h. After stirring, the solutions were pre-sheared by a Waring 7012S blender (Waring Products, Torrington, Connecticut, United States) at a rate of 16900 rpm for 35 s, followed by deoxidizing and sealing in brown opaque glass bottles for 12 h. The final polymer solutions were 2.5, 2, 1.75, 1.5, 1.0, and 0.5 kg/m^3^.

#### 2.1.3. Polymer Degradation Studies

**Figure 1:**
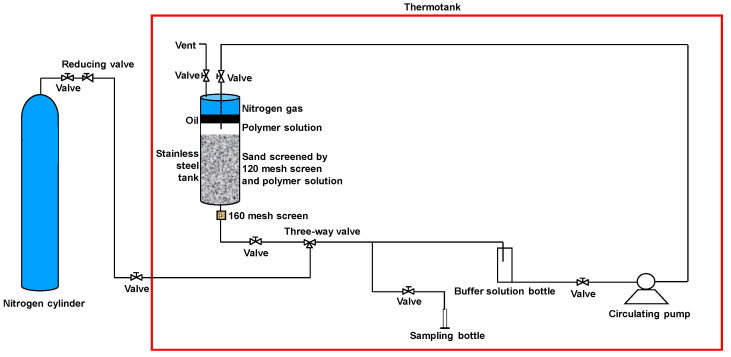
Schematic illustration of the polymer dynamic degradation experiment.

The static polymer degradation experiment began with 1.75 kg/m^3^ of polymer solution prepared by deoxidation and sealed in a stainless steel tank. The polymer solution was stored in a vacuum flask. Different polymer solutions were subjected to viscosity tests on the 1st, 7th, 15th, 30th, 45th, 65th, 90th, 105th, and 120th day. These tests were conducted at a consistent ambient temperature of 25 °C Compared with the polymer static degradation experiment, the polymer dynamic degradation experiment was more complicated, as shown in Figure 1. However, the temperature, sampling time, and sample viscosity measurements were the same for both experiments. An important step before the polymer dynamic degradation experiment was to immerse sand into 1.75 kg/m^3^ of polymer solution for two days. This could lead to a complete adsorption of the polymer, thereby eliminating the effect of polymer adsorption. Besides, oxygen was avoided during the polymer degradation experiments, in order to assure more accurate experimental results.

#### 2.1.4. Rheological Testing

Rheological testing was carried out on the Physica MCR-301 rheometer at 25 °C The rheology and viscosity of the polymer solution (1.75 kg/m^3^) and heavy oil were evaluated.

#### 2.1.5. Threshold Pressure Gradient Experiment

**Figure 2:**
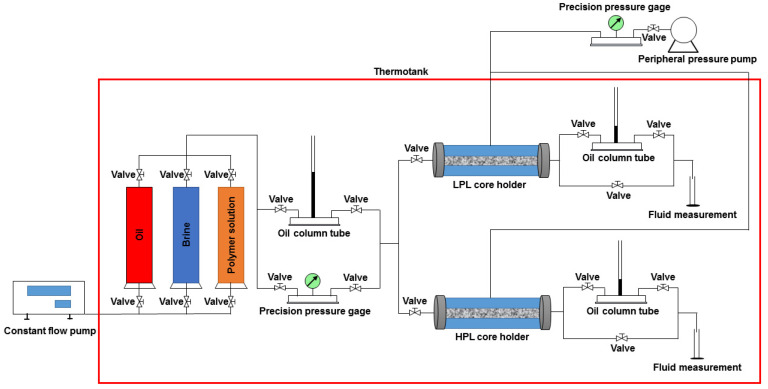
A schematic diagram of the threshold pressure gradient process.

The general workflow of the TPG process is presented in Figure 2. Considering that many factors can affect the TPG of heavy oil, especially permeability, this experiment not only measured the TPG of heavy oil in the low-permeability-layer (LPL) core but also in the high-permeability-layer (HPL) core. At first, it began with the TPG measurement of heavy oil in LPL core. When the temperature reached 25 °C the procedure of injecting 0.05 mL brine per minute was conducted to displace the heavy oil in the LPL core until achieving 4 times the pore volume (PV) of the LPL core. The LPL core was saturated by brine and set aside for 24 h. After that, the brine in the LPL core was displaced by injecting 0.05 mL heavy oil per minute. When the outlet water cut dropped below 2%, the flow rate increased 10 times until the end of water production. The oil column tubes were then put into use, and their oil column height was raised to about 5 cm and maintained for 12 h. Next, the height of the oil column in front of the LPL core holder was slowly and gradually increased, and the pressure gradient was the TPG of the heavy oil in the LPL core when the height of the oil column behind the LPL core holder started to rise. The TPG procedure of heavy oil in the HPL core was the same.

#### 2.1.6. PF Experiment

After TPG measurement, the PF experiment was subsequently performed. The oil column tubes were bypassed, and 1 mL of brine was injected every minute to carry out the displacement experiment, which lasted until the injection volume reached 1.4 PV. Following that, 1 mL of 1.75 kg/m^3^ polymer solution was injected every minute to displace the heavy oil, and it was stopped when the injection amount reached 0.36 PV. After 120 days, the displacement experiment was continued at the rate of 1 mL brine per minute until the injection volume reached 2.24 PV. Finally, the polymer system was sealed to avoid air inflow into the system.

### 2.2. Mathematical Models

#### 2.2.1. Model Assumptions

During the whole process of PF, only the water and oil phases were active, and no mass exchange was observed between these phases. The polymer solution consisted of both high- and low-molecular-weight components, and the former could determine the viscosity of the polymer solution. The fluids could be compressed, while the rocks not only could be compressed, but also could have anisotropy. The flow of fluids through the porous media involved an isothermal process, and the non-Newtonian flow was taken into account. Both capillary force and gravity were advised.

#### 2.2.2. Mechanism Models

PF is a complex process involving numerous parameter changes, which require mechanism models to describe. Here, the mathematical models for the main PF mechanism were derived from the published literature, as shown in Table 5.

Here, $\mu_{p}^{0}$ represents the viscosity of polymer solution at zero shear rate; $\mu_{w}$ denotes the water viscosity; $a_{1},$ $a_{2}$ and $a_{3}$ represent the parameters; $c_{p}$ represents the polymer concentration; $c_{s}$ represents the salt concentration; $s_{p}$ indicates the slope between $\left(\mu_{p}^{0}-\mu_{w}\right)/\mu_{w}$ and $c_{s}$ on a log-log plot. Under these conditions,$\;s_{p}$ is set to 0, and the possible effect of salt concentration is ignored. $\mu_{ps}$ represents the shear viscosity of the polymer solution; ${\dot{\gamma }}_{e}$ represents the equivalent shear rate; ${\dot{\gamma }}_{1/2}$ represents the shear rate at which the viscosity is equivalent to $\frac{\left(\mu_{p}^{0}+\mu_{w}\right)}{2},$ and $a_{4}$ represents a parameter. n denotes the flow behavior index, $v_{p}$ represents the Darcy velocity of the polymer solution, and k represents the permeability of rock; ϕ represents the porosity, and σ indicates the tortuosity of pores. In this case, σ is equivalent to 25/12. $c_{ap}$ represents the adsorbed concentration of polymer, $c_{apmax}$ represents the maximum adsorbed concentration of polymer, and $b_{p}$ represents the adsorption coefficient. $R_{k}$ indicates the water-phase permeability reduction factor, and RRF denotes the residual resistance factor. $f_{ipv}$ represents the inaccessible pore volume factor, $V_{i}$ represents the polymer inaccessible pore volume, and $V_{p}$ represents the pore volume. $R_{pd}$ denotes the first-order polymer degradation rate constant, d represents the derivative symbol, $c_{hp}$ represents the high-molecular-weight polymer concentration, and $t_{pd}$ represents the polymer degradation time.

#### 2.2.3. Equations

The flow equation of the water phase was constructed according to Darcy’s law [21], as shown below:

(1) $$\vec{\nu_{w}}=\frac{\vec{k}k_{rw}}{\mu_{wp}R_{k}}\nabla \Phi_{w}$$

where $\vec{\nu_{w}}$ represents the water-phase velocity tensor, $\vec{k}$ represents the absolute permeability tensor, $k_{rw}$ indicates the relative permeability of the brine, and $\mu_{wp}$ represents the viscosity of the water phase; ∇ represents the gradient operator, and $\Phi_{w}=p_{w}-\rho_{w}gD$; $p_{w}$ represents the pressure of the water phase, $\rho_{w}$ represents the density of the water phase, g denotes the gravitational acceleration, and D represents the vertical height.

Darcy’s law was further modified to describe the oil-phase flow due to heavy oil TPG [13]:

(2) $$\vec{\nu_{o}}=\{\begin{array}{c} \frac{\vec{k}k_{ro}}{\mu_{o}}\left(\nabla \Phi_{o}-G\right)\;\;\;if\;\nabla \Phi_{o}>G\;\\ 0\;\;\;\;\;\;\;\;\;\;\;\;\;\;if\;\nabla \Phi_{o}\leq G \end{array}$$

where $\vec{\nu_{o}}$ represents the oil-phase velocity tensor; $k_{ro}$ denotes the relative permeability of the oil phase; $\mu_{o}$ represents the viscosity of the oil phase; $\Phi_{o}=p_{o}-\rho_{o}gD$, $p_{o}$ and $\rho_{o}$ represent the pressure and density of the oil phase, respectively; and G represents the TPG of the oil phase.

The continuity equations for various components under standard ground conditions are listed below.

Continuity equation for water:

(3) $$\nabla \cdot \left(\frac{\vec{\nu_{w}}}{B_{w}}\right)+q_{w}\;=\;\frac{\partial }{\partial t}\left(\frac{\phi s_{w}}{B_{w}}\right)$$

Continuity equation for high-molecular-weight polymer:

(4) $$\nabla \cdot \left(\frac{\vec{\nu_{w}}c_{hp}}{B_{w}}\right)-c_{hp}R_{p}+q_{w}c_{hp}\;=\;\frac{\partial }{\partial t}\left[\frac{\phi \left(1-f_{ipv}\right)s_{w}c_{hp}}{B_{w}}\right]+\frac{\partial \left[\left(1-f_{ipv}\right)\left(1-\phi \right)\rho_{r}c_{ahp}\right]}{\partial t}$$

Continuity equation for low-molecular-weight polymer:

(5) $$\nabla \cdot \left(\frac{\vec{\nu_{w}}c_{lp}}{B_{w}}\right)+c_{hp}R_{p}+q_{w}c_{lp}\;=\;\frac{\partial }{\partial t}\left[\frac{\phi \left(1-f_{ipv}\right)s_{w}c_{lp}}{B_{w}}\right]+\frac{\partial \left[\left(1-f_{ipv}\right)\left(1-\phi \right)\rho_{r}c_{alp}\right]}{\partial t}$$

Continuity equation for oil:

(6) $$\nabla \cdot \left(\frac{\vec{\nu_{o}}}{B_{o}}\right)+q_{o}\;=\;\frac{\partial }{\partial t}\left(\frac{\phi s_{o}}{B_{o}}\right)$$

where $B_{w}$ and $B_{o}$ represent the formation volume factors of the water and oil phases, respectively; and $q_{w}$ and $q_{o}$ represent the source/sink terms. ∂ indicates partial derivatives, t represents time; $s_{w}$ and $s_{o}$ denote both water- and oil-phase saturations; $\rho_{r}$ represents the rock density. $c_{lp}$ represents the concentration of low-molecular-weight polymer; and $c_{ahp}$ and $c_{alp}$ represent the adsorption concentrations of the high- and low-molecular-weight polymers, respectively.

The above flow and continuity equations could describe the basic flow characteristics of water and oil phases. However, the relationship between some physical quantities in these phases must be additionally described by the auxiliary equation as well as the equations of state.

The following are the auxiliary equations:

(7) $$s_{w}+s_{o}=1$$

(8) $$p_{cow}\left(s_{w}\right)=p_{o}-p_{w}$$

where $p_{cow}\left(s_{w}\right)$ represents the capillary pressure in the water-oil system.

The equations of state are as follows:

(9) $$k_{ro}=k_{ro}\left(s_{w}\right)$$

(10) $$k_{rw}=k_{rw}\left(s_{w}\right)$$

(11) $$\rho_{o}=\rho_{o}\left(p_{o}\right)$$

(12) $$\rho_{w}=\rho_{w}\left(p_{w}\right)$$

(13) $$\phi =\phi \left(p_{r}\right)$$

where $p_{r}$ represents the reservoir pressure.

#### 2.2.4. Solution Method

It is necessary to ensure that the solutions obtained by these equations are unique. Therefore, the deterministic conditions are essential.

The following is the initial condition:

(14) $$p_{r}\left(x,y,z\right){|}_{t=0}=p_{ri}\left(x,y,z\right)$$

(15) $$s_{w}\left(x,y,z\right){|}_{t=0}=s_{wi}\left(x,y,z\right)$$

(16) $$c_{hp}\left(x,y,z\right){|}_{t=0}=c_{hpi}\left(x,y,z\right)$$

(17) $$c_{lp}\left(x,y,z\right){|}_{t=0}=c_{lpi}\left(x,y,z\right)$$

where (x,y,z) represents the coordinates; $p_{ri}$ denotes the initial reservoir pressure, $s_{wi}$ represents the initial water-phase saturation; and $c_{hpi}$ and $c_{lpi}$ indicate the initial concentrations of high- and low-molecular-weight polymers, respectively.

There are two kinds of boundary conditions: (i) internal boundary; and (ii) external boundary. The external boundary is a closed boundary:

(18) $$\frac{\partial p}{\partial n}{|}_{B}=f\left(x,y,z,t\right)=0$$

The formula, $\frac{\partial p}{\partial n}{|}_{B}$ represents the derivative of the boundary pressure in the direction of the outer normal. The following are the conditions for the internal boundary:

(19) $$Q_{w}\left(x,y,z,t\right){|}_{{\left(x,y,z\right)}_{well}}=Q_{w}\left(t\right)$$

(20) $$Q_{o}\left(x,y,z,t\right){|}_{{\left(x,y,z\right)}_{well}}=Q_{o}\left(t\right)$$

(21) $$c_{hp}\left(x,y,z,t\right){|}_{{\left(x,y,z\right)}_{well}}=c_{hp}\left(t\right)$$

(22) $$c_{lp}\left(x,y,z,t\right){|}_{{\left(x,y,z\right)}_{well}}=c_{lp}\left(t\right)$$

The $Q_{w}$ and $Q_{o}$ represent the flow rates of the water and oil phases, respectively. ${\left(x,y,z\right)}_{well}$ represents the grid coordinate of the well.

Continuity equations are categorized as partial differential equations, and they are difficult to solve using an analytic method [43]. Therefore, the finite difference method was chosen as an appropriate method to solve the problem. The following is the corresponding discrete form of the continuity equation.

Discrete form for water:

(23) $$\begin{array}{ll} {\left(T\lambda_{w}∆\Phi_{w}\right)}_{i+1/2,j,k}^{n+1} & -{\left(T\lambda_{w}∆\Phi_{w}\right)}_{i-1/2,j,k}^{n+1}+{\left(T\lambda_{w}∆\Phi_{w}\right)}_{i,j+1/2,k}^{n+1}\\ & -{\left(T\lambda_{w}∆\Phi_{w}\right)}_{i,j-1/2,k}^{n+1}+{\left(T\lambda_{w}∆\Phi_{w}\right)}_{i,j,k+1/2}^{n+1}\\ & -{\left(T\lambda_{w}∆\Phi_{w}\right)}_{i,j,k-1/2}^{n+1}+Q_{wi,j,k}^{n+1}\\ & =\left[{\left(\frac{v\phi s_{w}}{B_{w}}\right)}_{i,j,k}^{n+1}-{\left(\frac{v\phi s_{w}}{B_{w}}\right)}_{i,j,k}^{n}\right]/\Delta t \end{array}$$

Discrete form for high-molecular-weight polymer:

(24) $$\begin{array}{ll} {\left(T\lambda_{w}c_{hp}∆\Phi_{w}\right)}_{i+1/2,j,k}^{n+1} & -{\left(T\lambda_{w}c_{hp}∆\Phi_{w}\right)}_{i-1/2,j,k}^{n+1}+{\left(T\lambda_{w}c_{hp}∆\Phi_{w}\right)}_{i,j+1/2,k}^{n+1}\\ & -{\left(T\lambda_{w}c_{hp}∆\Phi_{w}\right)}_{i,j-1/2,k}^{n+1}+{\left(T\lambda_{w}c_{hp}∆\Phi_{w}\right)}_{i,j,k+1/2}^{n+1}\\ & -{\left(T\lambda_{w}c_{hp}∆\Phi_{w}\right)}_{i,j,k-1/2}^{n+1}-{\left(vc_{hp}R_{p}\right)}_{i,j,k}^{n+1}+{\left(Q_{w}c_{hp}\right)}_{i,j,k}^{n+1}\\ & =\{{\left[\frac{v\phi \left(1-f_{ipv}\right)s_{w}c_{hp}}{B_{w}}\right]}_{i,j,k}^{n+1}-{\left[\frac{v\phi \left(1-f_{ipv}\right)s_{w}c_{hp}}{B_{w}}\right]}_{i,j,k}^{n}\\ & +{\left[v\left(1-f_{ipv}\right)\left(1-\phi \right)\rho_{r}c_{ahp}\right]}_{i,j,k}^{n+1}\\ & -{\left[v\left(1-f_{ipv}\right)\left(1-\phi \right)\rho_{r}c_{ahp}\right]}_{i,j,k}^{n}\}/∆t \end{array}$$

Discrete form for low-molecular-weight polymer:

(25) $$\begin{array}{ll} {\left(T\lambda_{w}c_{lp}∆\Phi_{w}\right)}_{i+1/2,j,k}^{n+1} & -{\left(T\lambda_{w}c_{lp}∆\Phi_{w}\right)}_{i-1/2,j,k}^{n+1}+{\left(T\lambda_{w}c_{lp}∆\Phi_{w}\right)}_{i,j+1/2,k}^{n+1}\\ & -{\left(T\lambda_{w}c_{lp}∆\Phi_{w}\right)}_{i,j-1/2,k}^{n+1}+{\left(T\lambda_{w}c_{lp}∆\Phi_{w}\right)}_{i,j,k+1/2}^{n+1}\\ & -{\left(T\lambda_{w}c_{lp}∆\Phi_{w}\right)}_{i,j,k-1/2}^{n+1}+{\left(vc_{hp}R_{p}\right)}_{i,j,k}^{n+1}+{\left(Q_{w}c_{lp}\right)}_{i,j,k}^{n+1}\\ & =\{{\left[\frac{v\phi \left(1-f_{ipv}\right)s_{w}c_{lp}}{B_{w}}\right]}_{i,j,k}^{n+1}-{\left[\frac{v\phi \left(1-f_{ipv}\right)s_{w}c_{lp}}{B_{w}}\right]}_{i,j,k}^{n}\\ & +{\left[v\left(1-f_{ipv}\right)\left(1-\phi \right)\rho_{r}c_{alp}\right]}_{i,j,k}^{n+1}\\ & -{\left[v\left(1-f_{ipv}\right)\left(1-\phi \right)\rho_{r}c_{alp}\right]}_{i,j,k}^{n}\}/∆t \end{array}$$

Discrete form for oil:

(26) $$\begin{array}{ll} {\left(T\lambda_{o}∆\Psi_{o}\right)}_{i+1/2,j,k}^{n+1} & -{\left(T\lambda_{o}∆\Psi_{o}\right)}_{i-1/2,j,k}^{n+1}+{\left(T\lambda_{o}∆\Psi_{o}\right)}_{i,j+1/2,k}^{n+1}-{\left(T\lambda_{o}∆\Psi_{o}\right)}_{i,j-1/2,k}^{n+1}\\ & +{\left(T\lambda_{o}∆\Psi_{o}\right)}_{i,j,k+1/2}^{n+1}-{\left(T\lambda_{o}∆\Psi_{o}\right)}_{i,j,k-1/2}^{n+1}+Q_{oi,j,k}^{n+1}\\ & =\left[{\left(\frac{v\phi s_{o}}{B_{o}}\right)}_{i,j,k}^{n+1}-{\left(\frac{v\phi s_{o}}{B_{o}}\right)}_{i,j,k}^{n}\right]/\Delta t \end{array}$$

where n and (i,j,k) represent the time step number and grid block number, respectively. $T_{i+1/2,j,k}=\frac{2{\left(dydzk_{xx}\right)}_{i,j,k}{\left(dydzk_{xx}\right)}_{i+1,j,k}}{{\left(dydzk_{xx}\right)}_{i,j,k}dx_{i+1,j,k}+{\left(dydzk_{xx}\right)}_{i+1,j,k}dx_{i,j,k}}$, in which dx,dy, and dz represent the sizes of the grid blocks in x, y, and z directions, respectively. $k_{xx}$ represents the absolute permeability in x direction.

$$∆\Phi_{wi+1/2,j,k}=p_{wi+1,j,k}-p_{wi,j,k}-\frac{1}{2}\left(\rho_{wi+1,j,k}+\rho_{wi,j,k}\right)g\left(D_{i+1,j,k}-D_{i,j,k}\right)$$,

and

$$\lambda_{wi+1/2,j,k}=\{\begin{array}{c} {\left(\frac{k_{rw}}{\mu_{wp}B_{w}R_{k}}\right)}_{i+1,j,k}\;if\;∆\Phi_{wi+1/2,j,k}\geq 0\\ {\left(\frac{k_{rw}}{\mu_{wp}B_{w}R_{k}}\right)}_{i+1,j,k}\;if\;∆\Phi_{wi+1/2,j,k}<0 \end{array}$$

is in (Pa·s)^−1^.

$$∆\Psi_{oi+1/2,j,k}=\{\begin{array}{c} ∆\Phi_{0i+1/2,j,k}-{\left(Gd_{x}\right)}_{i+1/2,j,k}\;if\;∆\Phi_{0i+1/2,j,k}>{\left(Gd_{x}\right)}_{i+1/2,j,k}\\ 0\;if\;∆\Phi_{0i+1/2,j,k}\leq {\left(Gd_{x}\right)}_{i+1/2,j,k} \end{array}$$

where

$$∆\Phi_{oi+1/2,j,k}=p_{oi+1,j,k}-p_{oi,j,k}-\frac{1}{2}\left(\rho_{oi+1,j,k}+\rho_{oi,j,k}\right)g\left(D_{i+1,j,k}-D_{i,j,k}\right)$$

and

$${\left(Gd_{x}\right)}_{i+1/2,j,k}=\frac{1}{2}\left(G_{i+1,j,k}dx_{i+1,j,k}+G_{i,j,k}dx_{i,j,k}\right)$$

$Q_{wi,j,k}$ and $Q_{oi,j,k}$ represent the flow rates of the water and oil phases in (i,j,k) grid blocks, respectively. $v_{i,j,k}$ represents the volume of (i,j,k) grid blocks. Here, no other similar quantities are indicated.

To ensure computational stability, a fully implicit method was selected to solve the linear algebraic equations, which could be derived from Equations (23)–(26). The relevant calculations were derived from [44,45].

## 3. Results and Discussion

### 3.1. Polymer Degradation

**Figure 3:**
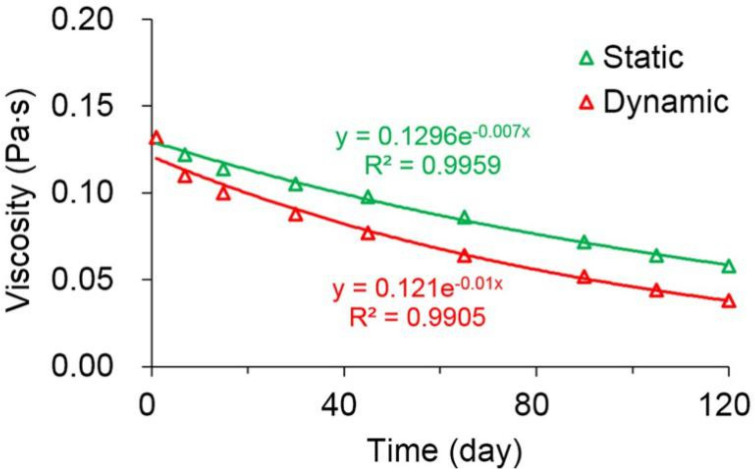
Static and dynamic degradation curves of the polymer solutions.

As shown in Figure 3, the static and dynamic degradation curves of polymer solutions were plotted based on their viscosity levels throughout the 120-day experiment. The viscosities of polymer solutions were exponentially correlated with time, and the square of correlation coefficient (R^2^) was more than 0.99. It has been previously reported that the viscosity of the polymer solution can decrease over time as the polymer long molecular chain breaks, thus resulting in a decrease in the average molecular weight of the polymer [31,32]. Combined with the following viscosity–concentration relationship, the first-order static and dynamic degradation rate constants of the polymer were calculated to be 0.003 and 0.005 day^−1^, respectively. The latter was greater due to the shear-induced breakage of long-molecular-chain polymers during the flow of polymer solution through the core [46].

### 3.2. Rheology of the Polymer Solution and Heavy Oil

**Figure 4:**
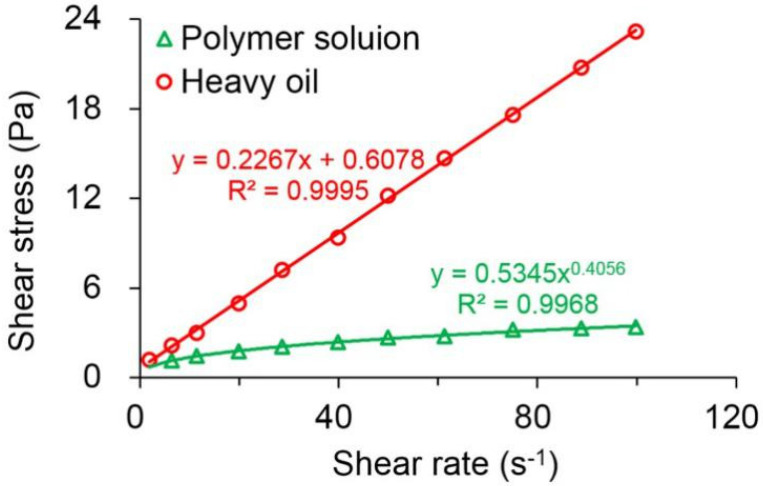
Rheological charts for the comparison between the polymer solution with a concentration of 1.75 kg/m^3^ and the heavy oil.

Figure 4 plots the rheological behavior of the polymer solution with a concentration of 1.75 kg/m^3^ and the heavy oil. By evaluating the relationship between the shear stress and shear rate of the polymer solution, it was found that the polymer exhibited a remarkable power law relationship, with an *R^2^* of above 0.99. Moreover, its flow behavior index was determined to be 0.4056, which corresponded to less than 1. This indicates that the polymer solution has a typical shear-thinning phenomenon. Unlike the polymer solution, the shear stress of the heavy oil displayed a good linear relationship with its shear rate, and the R^2^ was up to 0.9995. However, the line did not pass through the origin, which was in agreement with the rheological characteristics of Bingham fluid.

**Figure 5:**
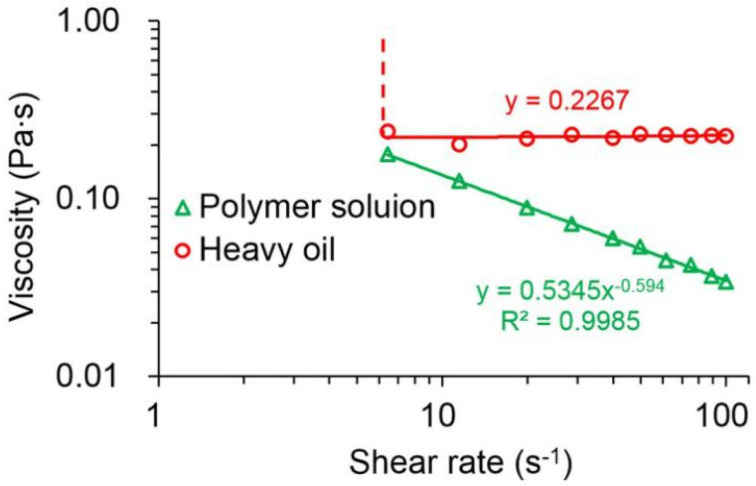
Viscosities of the polymer solution with a concentration of 1.75 kg/m^3^ and heavy oil at different shear rates.

Figure 5 shows the viscosities of polymer solution with a concentration of 1.75 kg/m^3^ and heavy oil at different shearing rates. From Figure 5, it can be clearly seen that the viscosity and the shear rate of the polymer solution demonstrated a remarkable power law relationship, and its viscosity decreased significantly with increasing shear rates. Its viscosity at 100 s^−1^ was 0.034 Pa·s, which was only one-fifth of that at 7.3 s^−1^. These results also indicate that the polymer solution has evident shear thinning. The polymer solution exhibits shear thinning, probably due to the fact that the entanglements between the polymer molecules are cracked at higher shear rates, which greatly shortens the hydrodynamic radius and reduces the viscosity of the polymer solution [47]. Different from the polymer solution, the viscosity of the heavy oil was infinite at low shear rates and remained at approximately 0.2267 Pa·s with increasing shear rates, which is consistent with Bingham fluid characteristics. The heavy oil exhibited Bingham fluid characteristics due to the solid-like nature of its network structure [48]. In other words, the heavy oil can only plastically deform with no flow under low stress. Under this low-stress condition, its network structure might not be destroyed. However, heavy oil will flow when the stress is greater than the yield stress.

**Figure 6:**
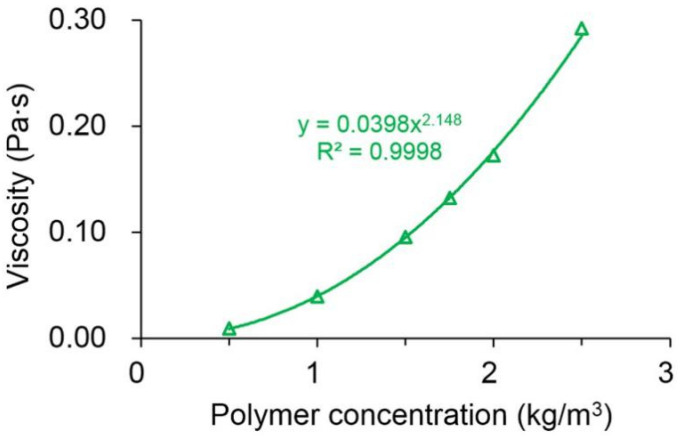
Relationship between the viscosity of the polymer solution and the polymer concentration.

In addition, the viscosity–concentration relationship of the polymer solutions is given in Figure 6. Notably, they demonstrated an excellent power law relationship, with an R^2^ of above 0.99. The viscosity of the polymer solution increased significantly with increasing polymer concentrations. The viscosity of the polymer solution with a concentration of 2.5 kg/m^3^ was 0.292 Pa·s, approximately 32 times greater than that with a concentration of 0.5 kg/m^3^. This is because the polymer solution with a higher polymer concentration has the longer polymer molecular chains and many more entanglements, thus resulting in an increase in the hydrodynamic radius as well as the viscosity of the polymer solution [49].

### 3.3. Threshold Pressure Gradient of Heavy Oil

According to the rheological properties of the heavy oil as discussed in the previous section, the heavy oil in this experiment was a Bingham fluid and could flow only when the stress exceeded the yield stress. Correspondingly, the heavy oil could flow only in the porous medium when the pressure gradient was greater than its TPG. The results of TPG experiments showed that the TPG values of heavy oil in LPL and HPL cores were 704 and 502 Pa/m, respectively. The TPG of heavy oil in the LPL core was greater than that in the HPL core, as a higher force is needed to overcome the greater resistance when it flows through rock at lower permeability [12].

### 3.4. Numerical Simulation

#### 3.4.1. Validation

**Figure 7:**
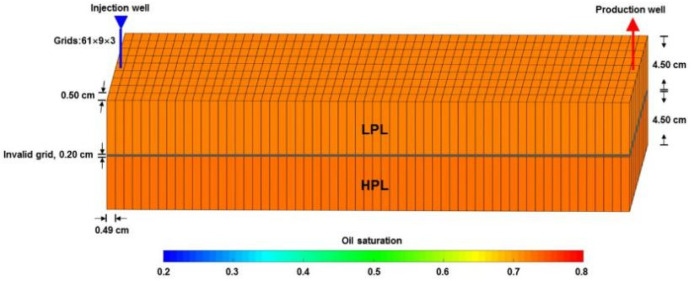
Grid system, well location, and 3D distributions of the initial oil saturation of the benchmark case.

**Figure 8:**
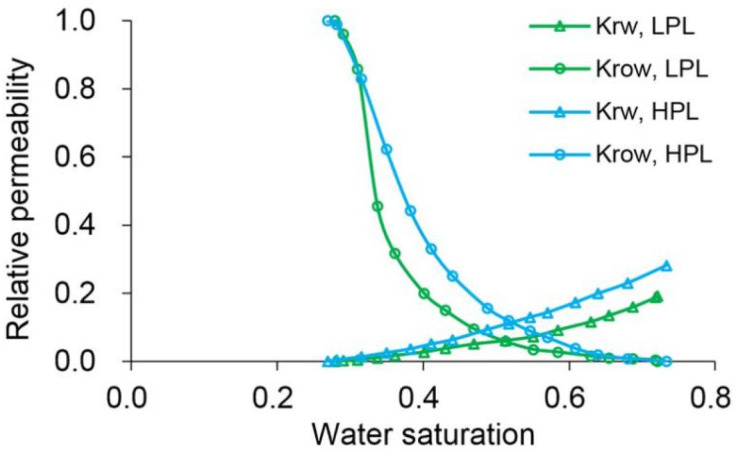
Relative permeabilities used in the benchmark case.

**Figure 9:**
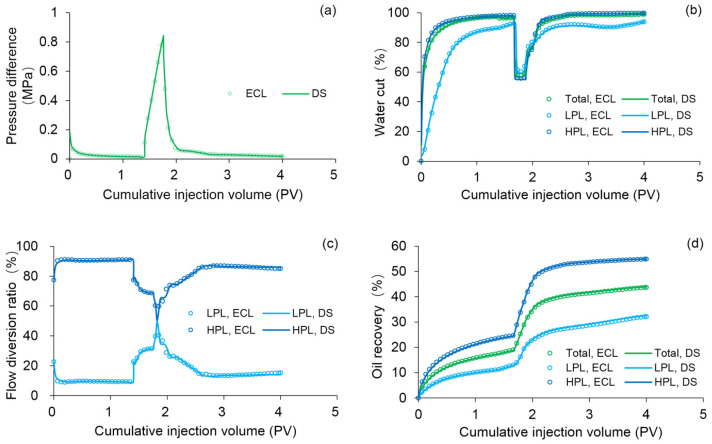
Comparison results for the (**a**) pressure difference, (**b**) water cut, (**c**) flow diversion ratio, and (**d**) oil recovery of the benchmark case simulated by ECL and DS methods.

**Figure 10:**
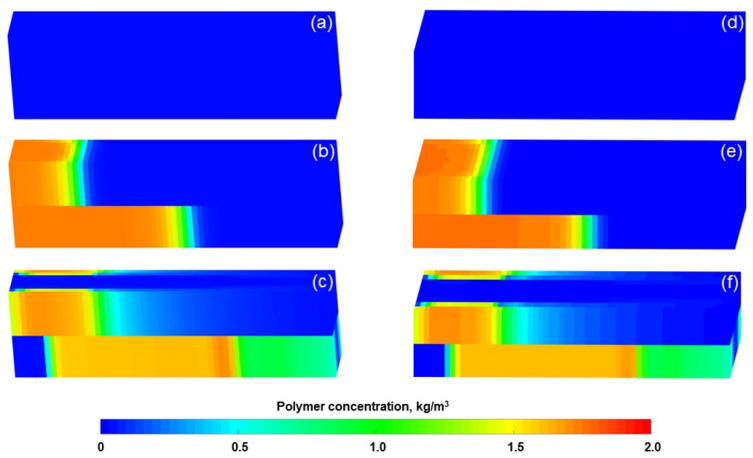
Three-dimensional polymer concentration distributions of the benchmark case after injection of (**a**) 1.4 PV initial water, (**b**) 0.36 PV polymer solution, and (**c**) 2.24 PV extended water, as revealed by the ECL analysis. Three-dimensional polymer concentration distributions of the benchmark case after injection of (**d**) 1.4 PV initial water, (**e**) 0.36 PV polymer solution, and (**f**) 2.24 PV extended water, as revealed by the DS analysis.

**Figure 11:**
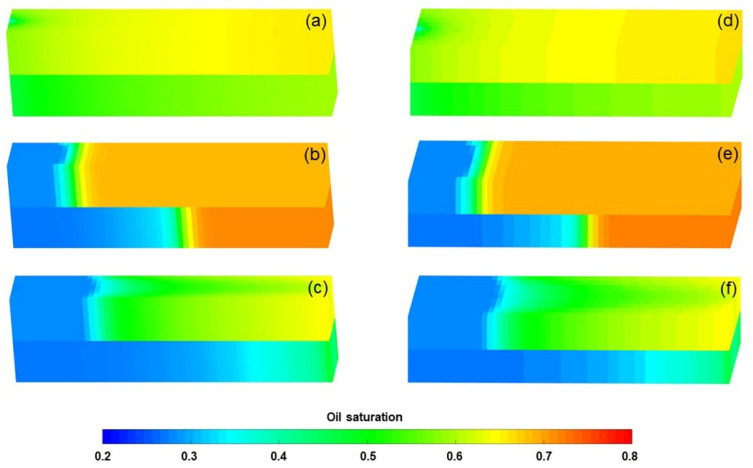
Three-dimensional oil saturation distributions of the benchmark case after injection of (**a**) 1.4 PV initial water, (**b**) 0.36 PV polymer solution, and (**c**) 2.24 PV extended water, as revealed by the ECL analysis. Three-dimensional oil saturation distributions of the benchmark case after injection of (**d**) 1.4 PV initial water, (**e**) 0.36 PV polymer solution, and (**f**) 2.24 PV extended water, as revealed by the DS analysis.

ECLIPSE V2013.1 (ECL) was used to simulate a benchmark case, and the obtained results were compared with those simulated by the designed simulator (DS). However, the specific situation of polymer degradation and non-Newtonian flow are not considered in the benchmark case. One reason is that although ECL is recognized as a commercial numerical reservoir simulator, it does not work well when some descriptions are needed for polymer degradation and non-Newtonian flow, especially TPG. Besides, this well-known software also has difficulty distinguishing between polymer static and dynamic degradation [25]. The main parameters of the benchmark case are summarized in Table 6 and Figure 7 and Figure 8. The comparison results for the main production indicators (such as the pressure differences, water cut, flow diversion ratio, and oil recovery), 3D polymer concentration, and oil saturation distribution of the benchmark case are shown in Figure 9, Figure 10 and Figure 11. The results of ECL and DS were relatively comparable. Thus, the validation of DS without considering polymer degradation and non-Newtonian flow was confirmed, manifesting a high degree of accuracy.

**Figure 12:**
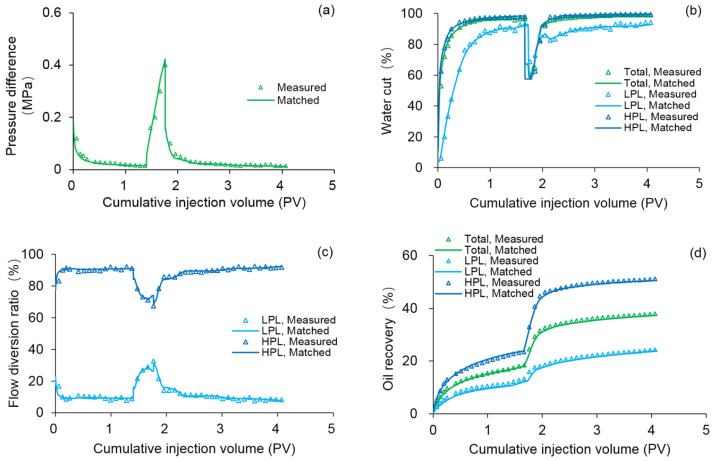
Polymer flooding experiment fitting results obtained by the DS in terms of: (**a**) pressure difference, (**b**) water cut, (**c**) flow diversion ratio, and (**d**) oil recovery.

**Figure 13:**
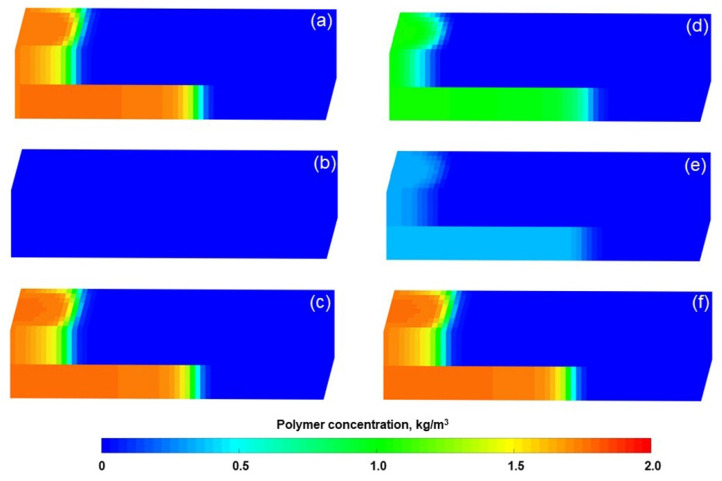
Three-dimensional concentration distributions of (**a**) high molecular weight, (**b**) low molecular weight, and (**c**) total polymers after injection of 0.36 PV polymer solution, and 3D concentration distributions of (**d**) high molecular weight, (**e**) low molecular weight, and (**f**) total polymers at 120 days after injection of 0.36 PV polymer solution, as revealed by the DS analysis of the polymer flooding experiment.

**Figure 14:**
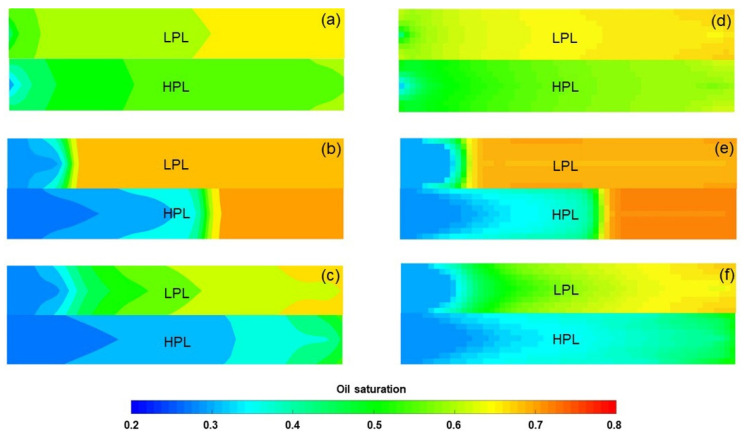
Actual oil saturation distributions of the LPL and HPL cores after injection of (**a**) 1.4 PV initial water, (**b**) 0.36 PV polymer solution, and (**c**) 2.24 PV extended water, as revealed by the saturation detector in the polymer flooding experiment. Oil saturation distributions of the LPL and HPL cores after injection of (**d**) 1.4 PV initial water, (**e**) 0.36 PV polymer solution, and (**f**) 2.24 PV extended water, as revealed by the DS analysis of the polymer flooding experiment.

To verify the accuracy of the DS considering polymer degradation and non-Newtonian flow, the PF experiment was fitted by DS, where the polymer static and dynamic degradation and the non-Newtonian flow of the polymer solution and heavy oil coexist. The parameters of the PF experiment fitting case differed from the benchmark case as shown in Table 7, and some other parameters were the same as those in the benchmark case. The fitting results of the main production indicators are presented in Figure 12. Notably, the difference among the production indicators was less than 1.6%. Figure 13 demonstrates the comparison results of polymer concentrations before and after the polymer solution was permitted to stand for 120 days. When 0.36 PV polymer solution was injected, the simulation results revealed the concentration of high-molecular-weight polymer near the injection well was 1.749 kg/m^3^, while that of low-molecular-weight polymer was 0.001 kg/m^3^. It is reasonable that these concentration changes are small due to the short duration of the polymer dynamic degradation (only 0.078 day). At 120 days after injection of 0.36 PV polymer solution, their concentrations became 1.12 kg/m^3^ and 0.63 kg/m^3^, respectively. The first-order degradation rate constant of the polymer solution was 0.003 day^−1^, which equals the static first-order degradation rate constant. It was obvious that the polymer only underwent static degradation during 120 days of the PF experiment, which was in line with the facts. Furthermore, their total polymer concentrations near the injection well did not change, and remained at about 1.75 kg/m^3^, consistent with the polymer theory. In addition, the comparison results for the actual oil saturation distributions of the LPL and HPL cores by the saturation detector in PF experiment and the oil saturation distributions of the LPL and HPL cores by the DS analysis are displayed in Figure 14. There are some slight differences between them, mainly due to the saturation detector, whose accuracy is affected by many factors such as laboratory conditions and experimental operations. Nevertheless, from the oil saturation distributions and its changing trends, the results can be considered similar. Therefore, the validation results of the DS with non-Newtonian flow and polymer degradation are shown to be positive.

#### 3.4.2. Effect of Polymer Degradation

**Figure 15:**
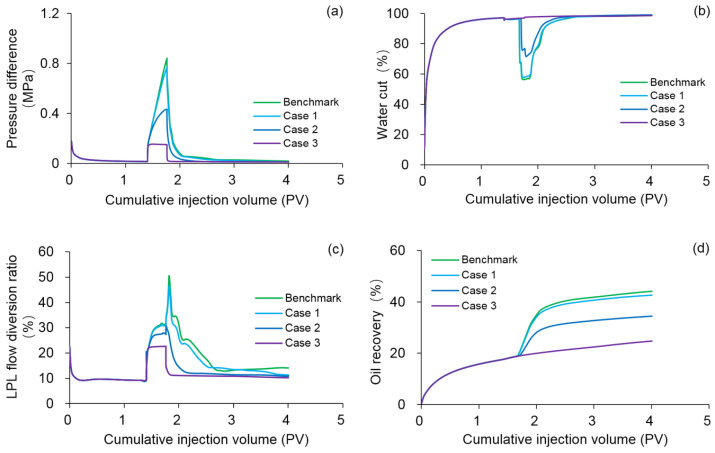
Comparison results of (**a**) pressure difference, (**b**) water cut, (**c**) LPL flow diversion ratio, and (**d**) oil recovery of the benchmark case and Cases 1–3.

**Figure 16:**
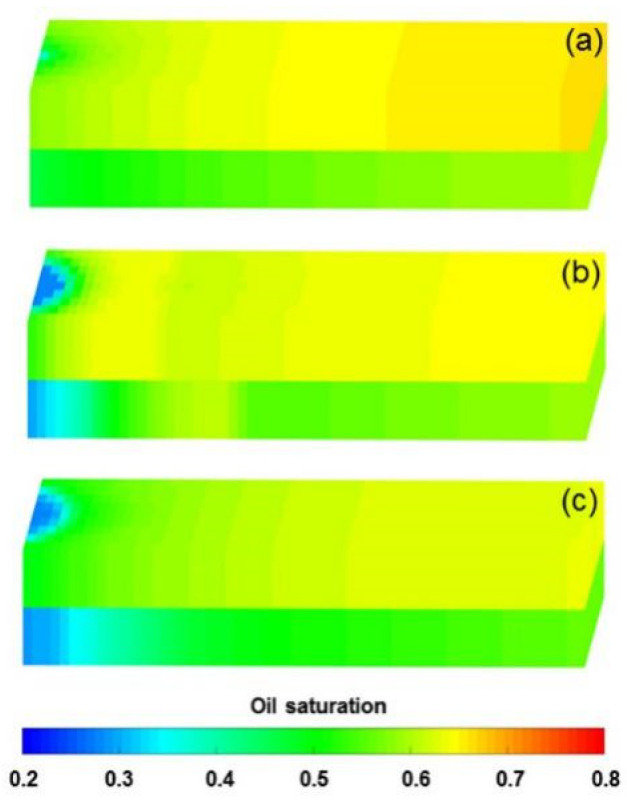
Three-dimensional oil saturation distributions of Case 3 after injection of (**a**) 1.4 PV initial water, (**b**) 0.36 PV polymer solution, and (**c**) 2.24 PV extended water.

To investigate the effect of polymer dynamic degradation on the production indicators and remaining oil saturation distribution, numerical simulations were conducted on Cases 1–3 by using the DS, where the first-order dynamic degradation rate constants were 0.001 day^−1^, 0.01 day^−1^, and 0.1 day^−1^, respectively, while other parameters were the same as those in the benchmark case. It has been suggested that a similar approach can be chosen to study the effects of polymer static degradation or both polymer static and dynamic degradation, but we did not carry it out here because the dynamic degradation of polymer mainly plays a significant role in actual PF. The comparison results of the benchmark case and Cases 1–3 are demonstrated in Figure 15, Table 8, and Figure 16. Evidently, the production indicators after injection of 1.4 PV initial water of the benchmark case and Cases 1–3 were the same. By comparing Figure 11b and 16a, it was observed that 3D oil saturation distributions after injection of 1.4 PV initial water of the benchmark case and Case 3 did not differ significantly. This might be attributed to the fact that no polymer was involved, and the polymer degradation did not have any effect on the simulation results. At the same time, this also reflects the computational stability and reliability of the DS. However, after injection of 0.36 PV polymer solution and 2.24 PV extended water, the pressure difference, LPL flow diversion ratio, and oil recovery declined, and the water cut increased with increasing polymer first-order dynamic degradation rate constant values. Furthermore, our results also demonstrated that the oil saturation in Case 3 was significantly higher than that in the benchmark case. The main reason for these phenomena is the degradation of polymer, which greatly reduces the viscosity of polymer solution. Such viscosity reduction leads to a significant increase in the mobility of water and oil phases, followed by a substantial reduction in the efficiency of PF, thus resulting in a significant increase in the remaining oil. After the completion of this research, the conclusion was that polymer degradation could have a significant negative impact on PF.

#### 3.4.3. Effect of Polymer Solution Shear Thinning

**Figure 17:**
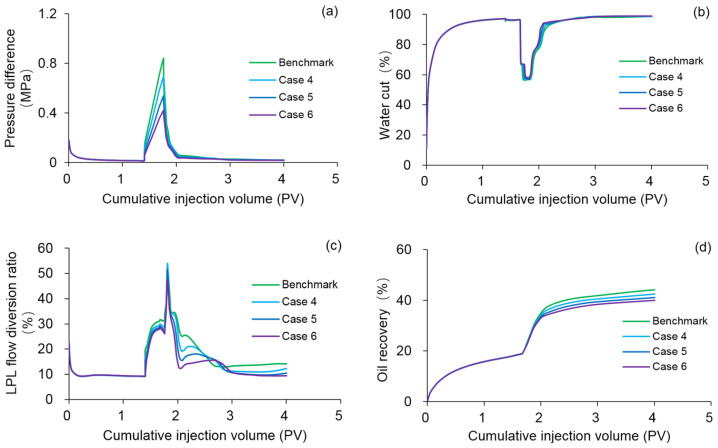
Comparison results for the (**a**) pressure difference, (**b**) water cut, (**c**) LPL flow diversion ratio, and (**d**) oil recovery of the benchmark case and Cases 4–6.

**Figure 18:**
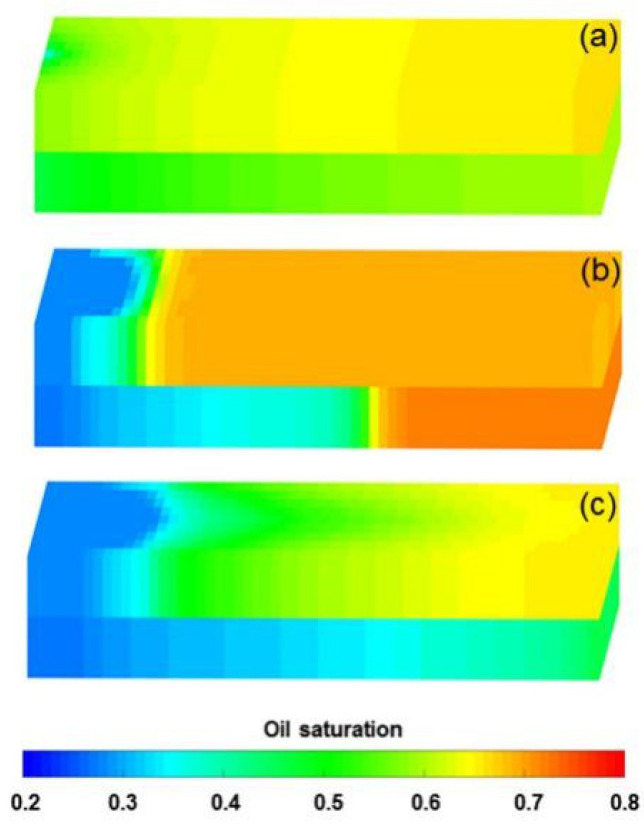
Three-dimensional oil saturation distributions of Case 6 after injection of (**a**) 1.4 PV initial water, (**b**) 0.36 PV polymer solution, and (**c**) 2.24 PV extended water.

Considering that the flow behavior index can explain the degree of deviation from Newtonian fluid and polymer solution shear thinning [50], numerical simulations were carried out on Cases 4–6 by using the DS, in order to study the effect of polymer solution shear thinning with flow behavior indexes of 0.5, 0.45, and 0.4, respectively. The other parameters were the same as those in the benchmark case. Figure 17 plots the comparison results of the main production indicators between the benchmark case and Cases 4–6. Table 8 lists the reduction in the production indicator of Cases 4–6 against the benchmark case, and Figure 18 shows the 3D oil saturation distributions of Case 6. Much like the effect of polymer degradation, the production indicators and remaining oil saturation distributions after injection of 1.4 PV initial water were relatively similar between the benchmark case and Cases 1–3 due to the absence of polymer solution. The pressure difference, LPL flow diversion ratio, and oil recovery decreased, while the water cut increased as the polymer solution flow behavior index decreased. Moreover, the oil saturation of Case 6 was significantly higher than that of the benchmark case, after injection of 0.36 PV polymer solution or 2.24 PV extended water. These results are attributed to the reduction in the viscosity of polymer solution caused by polymer solution shear thinning, which in turn increases the water-oil-phase mobility ratio and remaining oil. Thus, the polymer solution shear thinning can have adverse effects on PF performance.

#### 3.4.4. Effect of Heavy Oil Threshold Pressure Gradient

**Figure 19:**
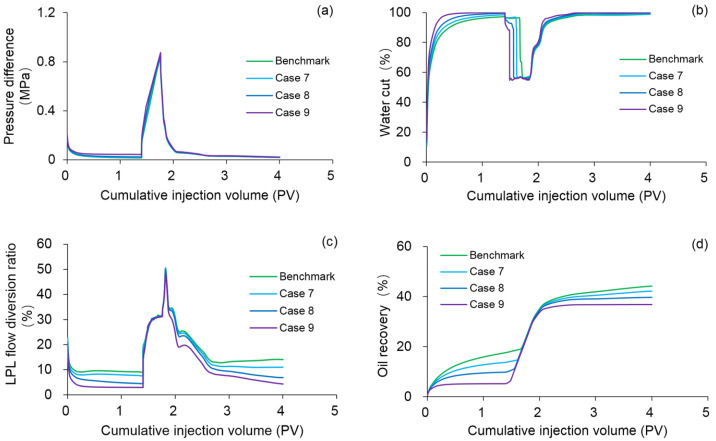
Comparison results for the (**a**) pressure difference, (**b**) water cut, (**c**) LPL flow diversion ratio, and (**d**) oil recovery of the benchmark case and Cases 7–9.

**Figure 20:**
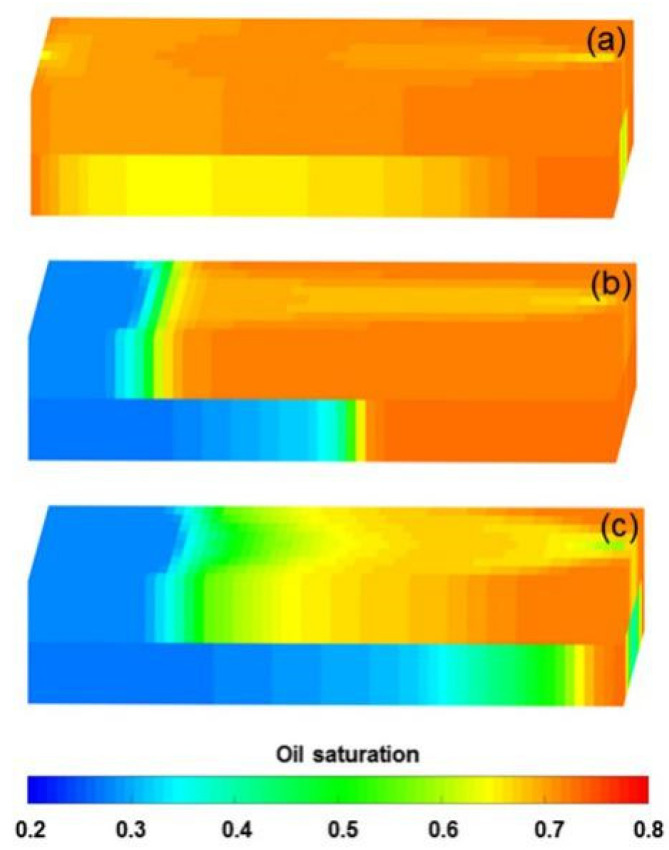
Three-dimensional oil saturation distributions of Case 9 after injection of (**a**) 1.4 PV initial water, (**b**) 0.36 PV polymer solution, and (**c**) 2.24 PV extended water.

To investigate the effect of heavy oil TPG on the production indicators and remaining oil saturation distribution, numerical simulations were performed on Cases 7–9 by using the DS, whose heavy oil TPGs were 5, 10, and 20 times higher compared to the PF experiment, respectively. Figure 19 plots the comparison results of the main production indicators between the benchmark case and Cases 7–9. Table 8 summarizes the reduction in the production indicator of Cases 7–9 against the benchmark case. Figure 20 shows 3D oil saturation distributions of Case 9. Notably, with increasing heavy oil TPG values, the pressure difference and water cut increased and the LPL flow diversion ratio and oil recovery decreased, after injection of 1.4 PV initial water. Moreover, the oil saturation of Case 9 was significantly higher than that of the benchmark case, after injection of 1.4 PV initial water. These results are mainly attributed to the flow difficulty of heavy oil caused by the heavy oil TPG. Unlike the results of 1.4 PV initial water injection, after injection of 0.36 PV polymer solution, the pressure difference and LPL flow diversion ratio increased and the water cut decreased, as the heavy oil TPG increased. In addition, a better polymer EOR was presented under the high heavy oil TPG condition, and the polymer EOR of Case 9 was approximately 11.4% more than that of the benchmark case. The presence of heavy oil TPG allowed PF to demonstrate its EOR capabilities and further prove its great significance in heavy oil reservoirs. However, after injection of 2.24 PV extended water, the increases in pressure difference and water cut and the decreases in LPL flow diversion ratio and oil recovery were observed with increasing heavy oil TPG values. Furthermore, the oil saturation of Case 9 was much higher than that of the benchmark case. The main reason for this result is that TPG increases the flow difficulty of heavy oil, thereby reducing the probability of PF to achieve the desired performance. Therefore, the heavy oil TPG can have a negative impact on PF.

## 4. Conclusions

In this work, both experiments and numerical simulations were conducted to study PF in heterogeneous heavy oil reservoirs. The experimental results demonstrated that the polymer first-order dynamic degradation rate constant was higher than the polymer first-order static degradation rate constant; the polymer solution had a typical shear thinning, and the heavy oil exhibited Bingham fluid characteristics; the flow of heavy oil in porous media was needed to overcome TPG. A novel and beneficial PF simulator was designed based on a new mathematical model, which provided a good description of polymer degradation and non-Newtonian flow, especially for distinguishing between the static and dynamic degradation of polymers as well as describing TPG. Such features fill the technological gap in current commercial simulators, which have not been able to give good estimations of polymer degradation and non-Newtonian flow and their effect on oil recovery during PF. The simulation results indicated that polymer degradation, polymer solution shear thinning, and heavy oil TPG all exerted a negative impact on PF performance. To reduce their negative effects, several approaches such as the application of more stable polymers with better performance and TPG reduction methods have been proposed [51,52,53,54,55,56]. In the near future, we will pay closer attention to these methods and attempt to study PF in heterogeneous heavy oil reservoirs based on a microscopic perspective, in order to enrich and deepen our research [57,58].

## Figures and Tables

**Table 1:** Typical properties of the polymer.

Properties	Description/Value
Type	Partially hydrolyzed polyacrylamide
Molecular weight	2.5 × 10^7^
Hydrolysis degree (%)	25.8
Solid content (wt%)	91.2
Filtration factor	1.4
Insoluble matter (wt%)	0.15
Dissolution rate (h)	<2
Granularity ≤ 0.2 mm (%)	2.6
Granularity ≥ 1.0 mm (%)	4.8

**Table 2:** Concentrations of the ion components in brine.

Ion Components	Concentration (mg/L)
Ca^2+^	48.92
Cl^−^	108.00
CO_3_^2−^	60.90
HCO_3_^−^	247.67
K^+^ and Na^+^	174.18
Mg^2+^	22.13
SO_4_^2−^	126.68
TDS	788.48

**Table 3:** Physical properties and SARA compositions of the heavy oil.

Parameters	Density (25 °C), kg/m^3^	Viscosity (25 °C), mPa∙s	Saturate, wt.%	Aromatic, wt.%	Resin, wt.%	Asphaltene, wt.%
Value	938.80	226.70	55.81	28.79	13.72	1.68

**Table 4:** Basic parameters of the cores.

Parameters	Core Name	
	Low-Permeability Layer	High-Permeability Layer
Height (cm)	4.5	4.5
Length (cm)	30	30
Width (cm)	4.5	4.5
Porosity (%)	25.4	26.0
Permeability (mD)	716	1820

**Table 5:** Mathematical models for the main PF mechanism.

References	Mathematical Model	Description
Flory [37], Sharafi et al. [26], Guo et al. [21]	$\mu_{p}^{0}=\mu_{w}\left[1+\left(a_{1}c_{p}+a_{2}c_{p}^{2}+a_{3}c_{p}^{3}\right)c_{s}{}^{s_{p}}\right]$	Polymer solution viscosity at the zero shear rate vs. polymer and salt concentrations.
Meter et al. [38], Wang et al. [35]	$\mu_{ps}=\mu_{w}+\frac{\mu_{p}^{0}-\mu_{w}}{1+{\left(\frac{{\dot{\gamma }}_{e}}{{\dot{\gamma }}_{1/2}}\right)}^{\left(a_{4}-1\right)}}$	Polymer solution viscosity vs. shear rate.
Zamani et al. [34]	${\dot{\gamma }}_{e}=4{\left(\frac{3n+1}{4n}\right)}^{\frac{n}{n-1}}\frac{v_{p}}{\sqrt{8k\phi \sigma }}$	Equivalent shear rate vs. Darcy velocity.
Adamson et al. [39], Chaudhuri et al. [40]	$c_{ap}=c_{apmax}\frac{b_{p}c_{p}}{1+b_{p}c_{p}}$	Polymer isothermal adsorption.
Bao et al. [41]	$R_{k}=1+\left(\mathrm{RRF}-1\right)\frac{c_{ap}}{c_{apmax}}$	Water-phase permeability reduction factor.
Hatzignatiou et al. [42], Sharafi et al. [25,26]	$f_{ipv}=\frac{V_{i}}{V_{p}}$	Polymer inaccessible pore volume factor.
Xin et al. [20,33]	$R_{p}=-\frac{dc_{hp}/c_{hp}}{dt_{p}}$	The first-order polymer degradation rate constant.

**Table 6:** The reservoir property, fluid property, initial conditions, and production data of the benchmark case.

Parameters	Value
Initial porosity of the LPL and HPL cores (fraction)	0.254, 0.26
Initial permeability of the LPL and HPL cores in x direction (mD)	716, 1820
Initial permeability of the LPL and HPL cores in y direction (mD)	716, 1820
Initial permeability of the LPL and HPL cores in z direction (mD)	71.6, 182
Reservoir temperature (°C)	25
Rock density of the LPL and HPL cores (kg/m^3^)	2600, 2570
Rock compressibility of the LPL and HPL cores (MPa^−1^)	2.78 × 10^−3^, 2.8 × 10^−3^
Stock tank oil density (kg/m^3^)	938.8
Initial oil viscosity (mPa∙s)	226.7
Oil compressibility (MPa^−1^)	1.16 × 10^−3^
Oil formation volume factor	1.066
Initial water density (kg/m^3^)	1
Water viscosity (mPa∙s)	0.69
Water compressibility (MPa^−1^)	4.26 × 10^−4^
Water formation volume factor	1.016
Polymer concentration (kg/m^3^)	1.75
Inaccessible pore volume factor of the LPL and HPL cores (fraction)	0.06, 0.05
Maximum polymer absorption of the LPL and HPL cores (kg/kg)	7.8 × 10^−5^, 6.9 × 10^−5^
Residual resistance factor of the LPL and HPL cores	3.4, 2.8
Initial reservoir pressure (MPa)	0
Initial water saturation of the LPL and HPL cores (fraction)	0.28, 0.27
Initial oil saturation of the LPL and HPL cores (fraction)	0.72, 0.73
Bottom hole pressure of production well (MPa)	0
Injection rate (mL/min)	1
Initial water injection volume (PV)	1.4
Polymer solution injection volume (PV)	0.36
Extended water injection volume (PV)	2.24

**Table 7:** Parameters of the polymer flooding experiment fitting case, which differ from the benchmark case.

Parameters	Value
TPG in the HPL and LPL cores (Pa/m)	469.45, 899.61
Flow behavior index of the polymer solution	0.4056
Time interval between polymer injection and subsequent water injection (day)	120
Polymer first-order static degradation rate constant (day^−1^)	0.003
Polymer first-order dynamic degradation rate constant (day^−1^)	0.005

**Table 8:** Reduction in the production indicators of Cases 1–9 against the benchmark case.

Production Indicators		Cases								
		1	2	3	4	5	6	7	8	9
After initial water flooding	Pressure difference (MPa)	0.00	0.00	0.00	0.00	0.00	0.00	−0.01	−0.01	−0.03
	Water cut (%)	0.00	0.00	0.00	0.00	0.00	0.00	−1.40	−2.42	−2.81
	LPL flow diversion (%)	0.00	0.00	0.00	0.00	0.00	0.00	1.58	4.76	6.22
	Oil recovery (%)	0.00	0.00	0.00	0.00	0.00	0.00	3.80	7.74	12.26
Before extended water flooding	Pressure difference (MPa)	0.07	0.41	0.69	0.15	0.30	0.42	−0.01	−0.02	−0.03
	Water cut (%)	−1.50	−20.06	−40.61	−0.29	−1.13	−2.53	0.36	0.54	0.74
	LPL flow diversion (%)	0.56	3.82	8.68	2.50	3.98	5.33	−0.07	−0.09	−0.17
	Oil recovery (%)	0.34	2.89	4.50	0.15	0.45	0.70	0.22	0.42	0.90
After extended water flooding	Pressure difference (MPa)	0.01	0.01	0.01	0.00	0.00	0.00	0.00	0.00	0.00
	Water cut (%)	−0.18	−0.22	−0.34	−0.05	−0.13	−0.24	−0.31	−0.96	−1.20
	LPL flow diversion (%)	3.88	4.41	4.45	1.77	3.71	4.74	3.09	7.26	9.76
	Oil recovery (%)	1.42	9.48	19.28	1.45	2.87	4.06	1.81	4.32	7.24

## Data Availability

Data sharing is not applicable to this article.
